# A vitamin-biomarker risk score for 90-day functional outcome after acute ischemic stroke: development and internal validation in a retrospective cohort

**DOI:** 10.3389/fneur.2026.1784930

**Published:** 2026-05-29

**Authors:** Yang Xu, Pengfei He, Xue Kang, Peihui Liu, Changming Xu

**Affiliations:** 1 Graduate Training Base, Jinzhou Medical University–Huludao Central Hospital, Huludao, Liaoning, China; 2 Department of Neurology and Interventional Vascular Surgery, Huludao Central Hospital, Huludao, Liaoning, China; 3 Department of Neurology I, Huludao Central Hospital, Huludao, Liaoning, China

**Keywords:** acute ischemic stroke, functional outcome, nomogram, prognosis, risk prediction, serum vitamin levels

## Abstract

**Background:**

The prognostic value of vitamin-related biomarkers in acute ischemic stroke (AIS) remains incompletely defined. This study evaluated associations between serum vitamin-related markers and 3-month functional outcome and developed an internally validated nomogram for individualized risk prediction.

**Methods:**

Consecutive AIS patients admitted to Huludao Central Hospital between November 2024 and July 2025 were retrospectively included (*n* = 655). The analyzed sample included 342 men (52.2%) and 313 women (47.8%), with a mean age of 66.84 ± 9.85 years and a mean admission NIHSS score of 3.98 ± 4.35. Poor outcome was defined as a 3-month modified Rankin Scale (mRS) score of 3–6, and good outcome as mRS ≤ 2. Demographic and clinical characteristics, routine laboratory indices, and serum levels of vitamin D, vitamin E, vitamin A, vitamin K1, vitamin B12, folate, and homocysteine were collected. Candidate variables were screened by univariable analyses and entered into multivariable logistic regression. Multivariable analyses adjusted for age, sex, BMI, admission NIHSS score, infarct location, vascular risk factors, atrial fibrillation, coronary heart disease, moderate-to-severe arterial stenosis, serum albumin, and total protein. A nomogram was constructed using the rms package in R. Internal validation was performed with 1,000 bootstrap resamples. Discrimination, calibration, and clinical net benefit were assessed using receiver operating characteristic curves, calibration curves, and decision curve analysis.

**Results:**

Among 655 patients, 346 (52.82%) had poor outcomes and 309 (47.18%) had good outcomes at 3 months. Multivariable analysis identified vitamin D, vitamin E, vitamin A, vitamin K1, vitamin B12, folate, and homocysteine as independent predictors of poor outcome. The nomogram showed good discrimination, with an AUC of 0.878 in the training set and 0.880 in the test set, together with favorable calibration and decision-curve performance. In the test set, both the vitamin-based model and the combined model outperformed the clinical baseline model (AUC 0.884 and 0.886 vs. 0.734; DeLong *p* < 0.001), whereas the combined model did not significantly improve over the vitamin-based model (ΔAUC = −0.005; *p* = 0.582).

**Conclusion:**

This serum vitamin-related biomarker nomogram may assist early risk stratification for 3-month functional outcome after AIS. However, as this was a single-center retrospective study with internal validation only, the model should be regarded as preliminary and requires external multicenter validation and prospective evaluation of clinical impact in patient management and outcomes before routine clinical use.

## 1 Introduction

Acute ischemic stroke (AIS) remains a leading cause of death and long-term disability worldwide (1, 2). Despite advances in reperfusion therapy, antithrombotic treatment, and secondary prevention, functional recovery after AIS varies widely across patients with apparently similar clinical presentations. Early risk stratification is therefore clinically important for treatment planning, complication surveillance, rehabilitation allocation, and communication with patients and families. Accordingly, there is continued interest in identifying objective, accessible, and clinically interpretable prognostic markers for AIS.

Nutritional and metabolic disturbances may contribute to outcome heterogeneity after AIS through effects on oxidative stress, inflammation, endothelial function, coagulation, and neurorepair (3). Several vitamin-related biomarkers, including vitamin D, vitamin E, vitamin A, vitamin K1, vitamin B12, folate, and homocysteine, have been associated with stroke risk or prognosis in prior studies (4–13). However, the literature remains difficult to translate into practice because individual biomarkers may be influenced by diet, supplementation, comorbidities, organ function, season, and acute-phase responses, and because prior studies have differed in patient selection, sampling windows, outcome definitions, and covariate adjustment. In addition, few studies have integrated multiple vitamin-related biomarkers into a single bedside prediction tool.

Nomograms are widely used in clinical prediction research because they combine multiple predictors into an individualized and interpretable risk estimate (14–16). In the present study, we evaluated the associations of serum vitamin-related biomarkers with 3-month functional outcome after AIS and developed an internally validated nomogram based on vitamin D, vitamin E, vitamin A, vitamin K1, vitamin B12, folate, and homocysteine. Our aim was to provide a practical biomarker-based tool for early prognostic stratification in routine clinical settings (17).

## 2 Materials and methods

### 2.1 General information (study design and participants)

This single-center retrospective cohort study consecutively included 655 patients with acute ischemic stroke (AIS) admitted to Huludao Central Hospital between November 2024 and July 2025. AIS was diagnosed on the basis of clinical presentation together with non-contrast cranial computed tomography (CT) and/or magnetic resonance imaging (MRI). All patients were admitted within 24 h after symptom onset.

The inclusion criteria were: (1) acute ischemic stroke (AIS) confirmed by cranial computed tomography (CT) or magnetic resonance imaging (MRI); (2) symptom onset-to-admission time ≤24 h; (3) serum vitamin-related biomarker results were available from the first routine venous blood sample obtained after admission, routinely within 24 h; and (4) a 3-month modified Rankin Scale (mRS) outcome was available.

Patients were excluded if they had infectious fever within 1 week prior to admission; regular use of vitamin supplements within 1 month prior to admission; comorbid malignant tumors, autoimmune diseases, severe anemia or other wasting diseases, or clinically significant gastrointestinal/malabsorption disorders that could substantially affect vitamin absorption or metabolism; or severe cardiac, hepatic, or renal insufficiency at the time of admission.

After 3 months had passed since the onset of the stroke, the modified Rankin Scale (mRS) score was the major outcome that was being studied. mRS ≤ 2 was considered to be a good outcome, while mRS 3–6 was considered to be a poor outcome.

### 2.2 Data collection and variable definitions

Data were extracted from the hospital electronic medical record system, laboratory information system, and imaging archive. Baseline variables included age, sex, body mass index (BMI), admission National Institutes of Health Stroke Scale (NIHSS) score, infarct location (anterior circulation, posterior circulation, or both), smoking status, alcohol consumption, hypertension, diabetes mellitus, coronary heart disease, atrial fibrillation, serum albumin, total serum protein, and vascular stenosis status.

Smoking and alcohol consumption were classified from the medical record as yes or no. Patients with any documented smoking history were categorized as smokers, and those with any documented alcohol consumption history were categorized as drinkers.

Routine laboratory parameters were extracted from the first blood tests performed after admission, including red blood cell count, hemoglobin, serum albumin, and total serum protein. Additional covariates that may influence both vitamin-related biomarkers and prognosis, including stroke subtype (e.g., TOAST classification), acute reperfusion treatment (intravenous thrombolysis and/or mechanical thrombectomy), onset-to-treatment time, inflammatory markers such as C-reactive protein, and detailed renal/liver function indices, were not consistently available in the retrospective database and therefore could not be reliably incorporated into the present multivariable model.

To improve confounding control within the limits of the available retrospective data, age, sex, BMI, admission NIHSS score, infarct location, vascular risk factors, atrial fibrillation, coronary heart disease, moderate-to-severe arterial stenosis, albumin, and total protein were considered in the clinical baseline or combined models. The remaining unmeasured variables are explicitly acknowledged as sources of residual confounding rather than ignored in interpretation.

### 2.3 Vascular assessment and stenosis grading

An evaluation of the patient’s vascular system was performed by means of a computed tomography angiography (CTA) of the head and neck while the patient was still in the hospital. According to the criteria that were used in North America, the degree of stenosis in the arteries was classified as follows: there was no stenosis (0%), mild stenosis (<50 %), moderate stenosis (50–69%), severe stenosis (70–99%), and occlusion (100%). The phrase “moderate-to-severe arterial stenosis” was defined in the current study as stenosis that was equivalent to or greater than 50 % (whether it was moderate stenosis, severe stenosis, or occlusion). This definition holds true regardless of the kind of stenosis.

### 2.4 Measurement of serum vitamin-related biomarkers

At Huludao Central Hospital, a high-volume regional demonstration stroke center in mainland China, vitamin-related biomarker testing is incorporated into the standardized admission laboratory evaluation for patients with acute ischemic stroke when no urgent clinical contraindication exists. Therefore, the biomarker data in this retrospective cohort were not obtained from stored research samples or post-hoc biobanking; they were extracted retrospectively from routine clinical laboratory records generated during standard inpatient care.

For each included patient, venous blood was collected as part of the first admission blood sampling, routinely within 24 h after admission, and submitted to the hospital clinical laboratory under standard operating procedures. Serum biomarkers included vitamin D, vitamin E, vitamin A, vitamin K1, vitamin B12, folate, and homocysteine. Because the study was retrospective, only patients with complete routine biomarker results and available 3-month mRS outcomes were included; no additional blood draw was performed for research purposes.

The unit for vitamin B12 was recorded as pg./mL, consistent with the original laboratory report. Assay-manufacturer information, between-run coefficients of variation, and instrument-level calibration records were not uniformly retrievable for all patients and are acknowledged as potential sources of measurement variability.

### 2.5 Handling of missing data

For variables with <5% missingness, complete-case analysis was used. If missingness had exceeded 5% or involved key predictors, multiple imputation with 10 imputations would have been performed using available covariates. In the present dataset, variables used for model development and model comparison were complete; therefore, imputation was not required.

### 2.6 Follow-up and outcome assessment

The primary outcome was the 3-month modified Rankin Scale (mRS) score after stroke onset. Follow-up was scheduled at approximately 90 days after discharge/stroke onset, with an allowable time window of ±7 days. Follow-up assessments were primarily conducted during outpatient stroke clinic visits. Patients who did not attend scheduled visits were assessed by telephone interview, and information from the main caregiver was used when necessary. The mRS was evaluated using a standardized interview protocol by neurologists or research staff trained in mRS assessment. Whenever feasible, outcome assessors were blinded to baseline vitamin measurements to reduce information bias. Follow-up data were double-checked and underwent logical consistency checks. All 655 enrolled patients had available 3-month mRS outcomes, with no loss to follow-up.

### 2.7 Statistical analysis and model development

Multivariable modeling was performed with attention to both clinical relevance and statistical stability. Candidate clinical covariates were selected *a priori* from established prognostic factors and potential confounders available in the retrospective database, including age, sex, BMI, admission NIHSS score, infarct location, vascular risk factors, atrial fibrillation, coronary heart disease, moderate-to-severe arterial stenosis, albumin, and total protein. Vitamin-related biomarkers were then evaluated in logistic regression models with 3-month outcome as the dependent variable (0 = good outcome, 1 = poor outcome). Adjusted odds ratios (ORs) with 95% confidence intervals (CIs) were used to quantify associations, and multicollinearity was assessed using variance inflation factors (VIFs), with VIF < 5 considered acceptable. Receiver operating characteristic (ROC) curves were used to evaluate individual biomarkers, and AUCs, sensitivity, specificity, and optimal cutoffs were reported. Given 346 poor-outcome events and 7 final predictors, the events-per-variable ratio was 49.4, supporting model stability.

Model development followed a prespecified reproducible workflow. The full cohort was randomly divided, using stratification by 3-month outcome and a fixed random seed of 322, into a training set (70%) and an independent test set (30%). All preprocessing steps were performed using the training set only. The vitamin-based model included the seven biomarkers that remained independently associated with outcome in multivariable analysis: homocysteine, folate, vitamin B12, vitamin K1, vitamin A, vitamin E, and vitamin D. A nomogram was then constructed in the training set using the rms package in R. After model fitting, coefficients were fixed and applied unchanged to the test set to generate predicted probabilities, thereby avoiding model refitting in the validation sample.

For reproducibility, the final vitamin-based model was expressed as the following logistic equation: logit(Poor outcome) = 11.614–0.207 × homocysteine + 0.164 × folate − 0.007 × vitamin B12 – 1.328 × vitamin K1–0.063 × vitamin A − 0.356 × vitamin E + 0.105 × vitamin D. The corresponding predicted probability was calculated as *p* = 1/[1 + exp.(−LP)], where LP denotes the linear predictor. The full set of regression coefficients, the intercept, adjusted odds ratios, and 95% confidence intervals are reported to facilitate independent reproduction, external validation, and future model updating. Because the nomogram is a graphical transformation of this fitted logistic model, predicted probabilities can be reproduced directly from the published equation and coefficients.

Discrimination was quantified by the C-index/AUC, with 95% CIs estimated using 1,000 bootstrap resamples. Calibration was assessed with bootstrap calibration curves (1,000 resamples) and the Hosmer–Lemeshow goodness-of-fit test (*g* = 10) in both the training and test sets. Decision curve analysis evaluated clinical net benefit across threshold probabilities from 0.01 to 0.99. These procedures, together with use of an independent held-out test set, were intended to reduce optimism and support internal validation.

To assess incremental predictive value, we additionally developed two comparator logistic regression models using the same train/test split and evaluation workflow: (1) a clinical baseline model including age, sex, BMI, admission NIHSS score, infarct location, smoking, alcohol consumption, hypertension, diabetes mellitus, coronary heart disease, atrial fibrillation, moderate-to-severe arterial stenosis (≥50%), serum albumin, and total protein; and (2) a combined model incorporating these clinical variables plus the seven vitamin-related biomarkers. AUCs were compared using DeLong’s test. All statistical tests were two-sided, and *p* < 0.05 was considered statistically significant. These analyses were intended to assess whether the biomarker panel added predictive information beyond routinely available clinical variables, rather than to establish causality or justify treatment decisions based on biomarker levels alone.

### 2.8 Ethics statement

The study protocol was approved by the Ethics Committee of Huludao Central Hospital (approval number: KY2025-31). Because this was a retrospective observational study based on de-identified data obtained during routine clinical care and no additional blood sampling or intervention was performed for research purposes, the requirement for written informed consent was waived by the Ethics Committee. The study was conducted in accordance with the principles of the Declaration of Helsinki.

## 3 Results

### 3.1 Outcome grouping and univariable comparisons

A total of 655 patients with AIS were included. At 3 months, 309 patients (47.18%) had a good outcome (mRS ≤ 2) and 346 (52.82%) had a poor outcome (mRS 3–6). No patient was lost to follow-up.

Univariable comparisons showed significant between-group differences in several baseline clinical characteristics and laboratory indices (all *p* < 0.05; Table 1). Compared with patients with good outcomes, those with poor outcomes were older (67.98 ± 9.96 vs. 65.57 ± 9.58 years, *p* = 0.002), had higher admission NIHSS scores (4.65 ± 4.82 vs. 3.24 ± 3.62, *p* < 0.001), and more often had moderate-to-severe arterial stenosis, hypertension, diabetes mellitus, coronary heart disease, atrial fibrillation, smoking history, and alcohol consumption (all *p* < 0.05).

**Table 1:** Univariable comparison of baseline characteristics between outcome groups [*n* (%) or mean ± SD].

Variable	Poor outcome (*n* = 346)	Good outcome (*n* = 309)	*χ*^2^/*t*	*p* value
Sex			0.134	0.714
Male	183 (52.9)	159 (51.5)		
Female	163 (47.1)	150 (48.5)		
Age (years)	67.98 ± 9.96	65.57 ± 9.58	−3.145	0.002
Body mass index (BMI)	20.17 ± 4.00	19.19 ± 3.64	−3.267	0.001
NIHSS score at admission	4.65 ± 4.82	3.24 ± 3.62	−4.207	<0.001
Smoking history			12.240	<0.001
Yes	177 (51.2)	116 (37.5)		
No	169 (48.8)	193 (62.5)		
Alcohol use			8.400	0.004
Yes	156 (45.1)	105 (34.0)		
No	190 (54.9)	204 (66.0)		
History of hypertension			5.505	0.019
Yes	193 (55.8)	144 (46.6)		
No	153 (44.2)	165 (53.4)		
History of diabetes mellitus			11.181	<0.001
Yes	149 (43.1)	94 (30.4)		
No	197 (56.9)	215 (69.6)		
History of coronary heart disease			9.889	0.002
Yes	157 (45.4)	103 (33.3)		
No	189 (54.6)	206 (66.7)		
History of atrial fibrillation			4.626	0.031
Yes	61 (17.6)	36 (11.7)		
No	285 (82.4)	273 (88.3)		
Moderate-to-severe arterial stenosis (≥50%)			4.406	0.036
Yes	137 (39.6)	98 (31.7)		
No	209 (60.4)	211 (68.3)		
Infarct location			5.967	0.051
Anterior circulation	275 (79.5)	267 (86.4)		
Posterior circulation	63 (18.2)	39 (12.6)		
Both anterior and posterior	8 (2.3)	3 (1.0)		
Red blood cell count (×10^12^/L)	4.67 ± 0.57	4.61 ± 0.53	−1.390	0.165
Hemoglobin (g/L)	139.10 ± 17.33	140.63 ± 16.07	1.167	0.244
Serum albumin (g/L)	40.44 ± 3.49	41.28 ± 3.82	2.917	0.004
Total serum protein (g/L)	64.37 ± 4.41	65.25 ± 6.29	2.055	0.040
Homocysteine (μmol/L)	11.01 ± 2.52	12.93 ± 2.66	9.427	<0.001
Folate (ng/mL)	6.43 ± 1.57	5.76 ± 1.92	−4.914	<0.001
Vitamin B12 (pg/mL)	441.39 ± 81.41	528.57 ± 84.84	13.412	<0.001
Vitamin K1 (ng/mL)	0.74 ± 0.41	1.07 ± 0.52	9.002	<0.001
Vitamin A (μg/dL)	28.72 ± 9.35	38.26 ± 11.35	11.649	<0.001
Vitamin E (mg/L)	11.60 ± 2.38	14.13 ± 1.98	14.877	<0.001
Vitamin D (ng/mL)	14.16 ± 2.20	13.24 ± 2.96	−4.504	<0.001

NIHSS, National Institutes of Health Stroke Scale; SD, standard deviation.

Patients with poor outcomes also had lower serum albumin and total protein levels and different distributions of several vitamin-related biomarkers (all *p* < 0.05; Table 1). Specifically, vitamin D and folate levels were higher in the poor-outcome group, whereas vitamin E, vitamin A, vitamin K1, vitamin B12, and homocysteine also differed significantly between groups. These univariable findings should be interpreted cautiously and not as evidence of causality.

Notably, although higher serum vitamin D and folate levels were observed in patients with poor outcomes in this cohort, these associations should be interpreted with caution because they were observational and may reflect residual confounding, reverse causation, acute-phase biological shifts, prior dietary or supplement exposure, measurement variability, or non-linear relationships rather than a direct harmful effect of these micronutrients.

### 3.2 Multivariable logistic regression analysis

In the multivariable logistic regression model, homocysteine, folate, vitamin B12, vitamin K1, vitamin A, vitamin E, and vitamin D remained independently associated with poor 3-month outcome (all *p* < 0.05; Table 2). Table 2 reports the regression coefficients, adjusted odds ratios, 95% confidence intervals, and intercept required to reproduce the fitted model.

**Table 2:** Full multivariable logistic regression model for poor 3-month outcome.

Variable	β	SE	Wald χ^2^	*p* value	OR	95% CI
Age	−0.002	0.014	0.025	0.875	0.998	0.971–1.025
BMI	−0.025	0.035	0.494	0.482	0.976	0.911–1.045
NIHSS at admission	0.016	0.027	0.357	0.550	1.016	0.964–1.071
Smoking history	0.309	0.219	1.994	0.158	1.362	0.887–2.091
Alcohol use	0.387	0.222	3.039	0.081	1.472	0.953–2.275
Hypertension	0.275	0.220	1.554	0.213	1.316	0.854–2.028
Diabetes mellitus	0.196	0.227	0.750	0.386	1.217	0.780–1.897
Coronary heart disease	0.306	0.225	1.843	0.175	1.358	0.873–2.111
Atrial fibrillation	0.516	0.306	2.839	0.092	1.676	0.919–3.056
Moderate-to-severe stenosis (≥50%)	0.316	0.228	1.918	0.166	1.371	0.877–2.144
Serum albumin	0.045	0.031	2.070	0.150	1.046	0.984–1.111
Total serum protein	−0.034	0.020	2.969	0.085	0.967	0.930–1.005
Homocysteine	−0.207	0.049	17.467	<0.001	0.813	0.738–0.896
Folate	0.164	0.071	5.346	0.021	1.178	1.025–1.354
Vitamin B12	−0.007	0.001	22.217	<0.001	0.993	0.990–0.996
Vitamin K1	−1.328	0.295	20.320	<0.001	0.265	0.149–0.472
Vitamin A	−0.063	0.012	29.713	<0.001	0.939	0.918–0.960
Vitamin E	−0.356	0.064	31.383	<0.001	0.700	0.618–0.793
Vitamin D	0.105	0.047	4.861	0.027	1.110	1.012–1.219
Intercept	11.614	2.345	24.536	<0.001		

Outcome was coded as 1 = poor outcome (mRS 3–6) and 0 = good outcome (mRS ≤2). OR, odds ratio; CI, confidence interval; SE, standard error. The intercept and β coefficients can be used to reproduce predicted probabilities from the logistic model; OR and 95% CI are not applicable to the intercept.

### 3.3 ROC analyses of individual biomarkers

**Figure 1:**
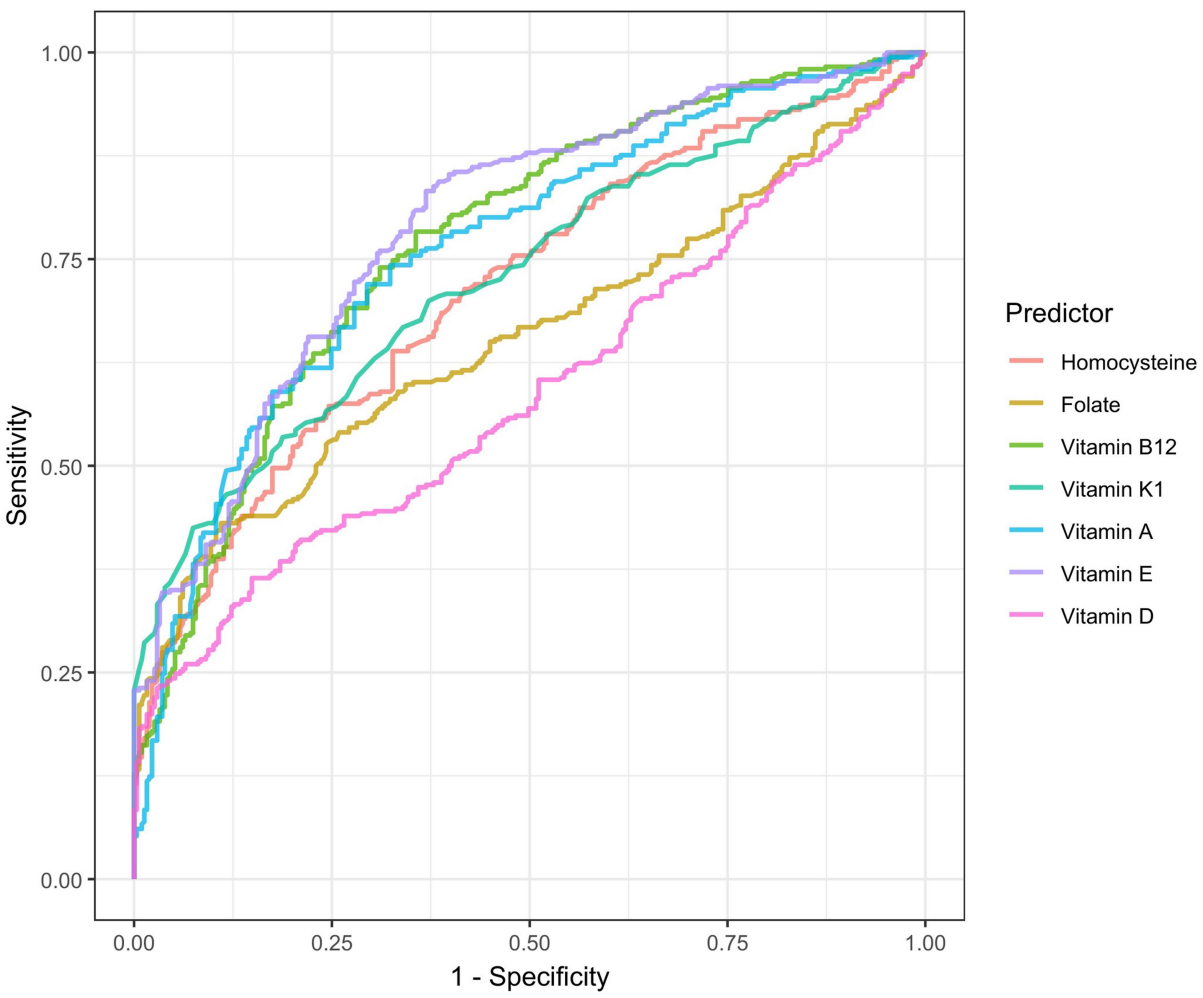
ROC curves of individual biomarkers for predicting poor 3-month outcome.

Receiver operating characteristic (ROC) analyses were performed for the seven biomarkers that remained significant in the multivariable model (Figure 1; Table 3). The AUCs for predicting poor outcomes were 0.713 for homocysteine, 0.655 for folate, 0.771 for vitamin B12, 0.728 for vitamin K1, 0.763 for vitamin A, 0.789 for vitamin E, and 0.590 for vitamin D. The optimal cutoff values were 11.110, 4.960, 486.710, 0.580, 31.840, 13.690, and 12.023, respectively.

**Table 3:** ROC performance of independent predictors (AUC, cutoffs, sensitivity, and specificity).

Marker	AUC	95% CI	Cutoff	Sensitivity (%)	Specificity (%)
Homocysteine	0.713	0.674–0.751	11.110	54.0	78.6
Folate	0.655	0.612–0.696	4.960	43.1	89.0
Vitamin B12	0.771	0.734–0.808	486.710	74.0	68.9
Vitamin K1	0.728	0.688–0.765	0.580	42.5	92.6
Vitamin A	0.763	0.726–0.799	31.840	72.0	70.6
Vitamin E	0.789	0.754–0.824	13.690	83.2	63.1
Vitamin D	0.590	0.545–0.632	12.023	36.4	85.1

AUC, area under the ROC curve; CI, confidence interval; ROC, receiver operating characteristic.

Among the individual biomarkers, vitamin E (AUC = 0.789) and vitamin B12 (AUC = 0.771) showed relatively better discriminative performance, whereas vitamin D demonstrated limited discrimination (AUC = 0.590) (Table 3).

### 3.4 Nomogram for predicting poor outcomes in patients with acute ischemic stroke

**Figure 2:**
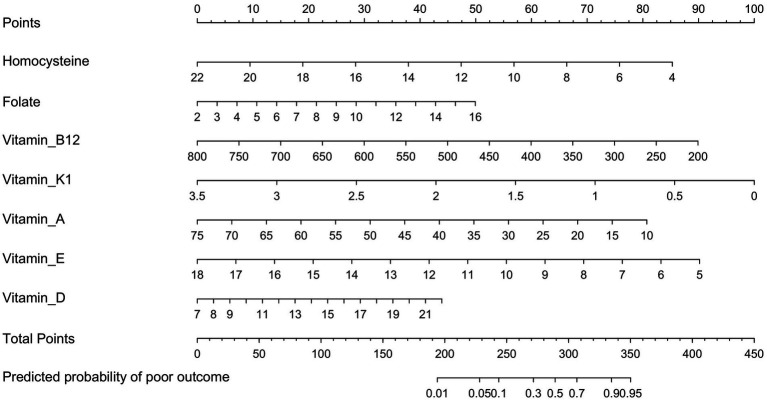
Nomogram for predicting poor 3-month outcome in AIS.

A nomogram was constructed from the seven independent predictors identified in the multivariable logistic regression model: homocysteine, folate, vitamin B12, vitamin K1, vitamin A, vitamin E, and vitamin D (Figure 2).

To apply the nomogram, the value of each predictor is projected to the corresponding points scale, the points are summed, and the total score is converted into an individualized predicted probability of poor 3-month outcome. Because the nomogram is a graphical representation of the underlying logistic model, reporting the full equation alongside the nomogram would further facilitate independent validation and future model updating.

### 3.5 Performance of the nomogram model

**Figure 3:**
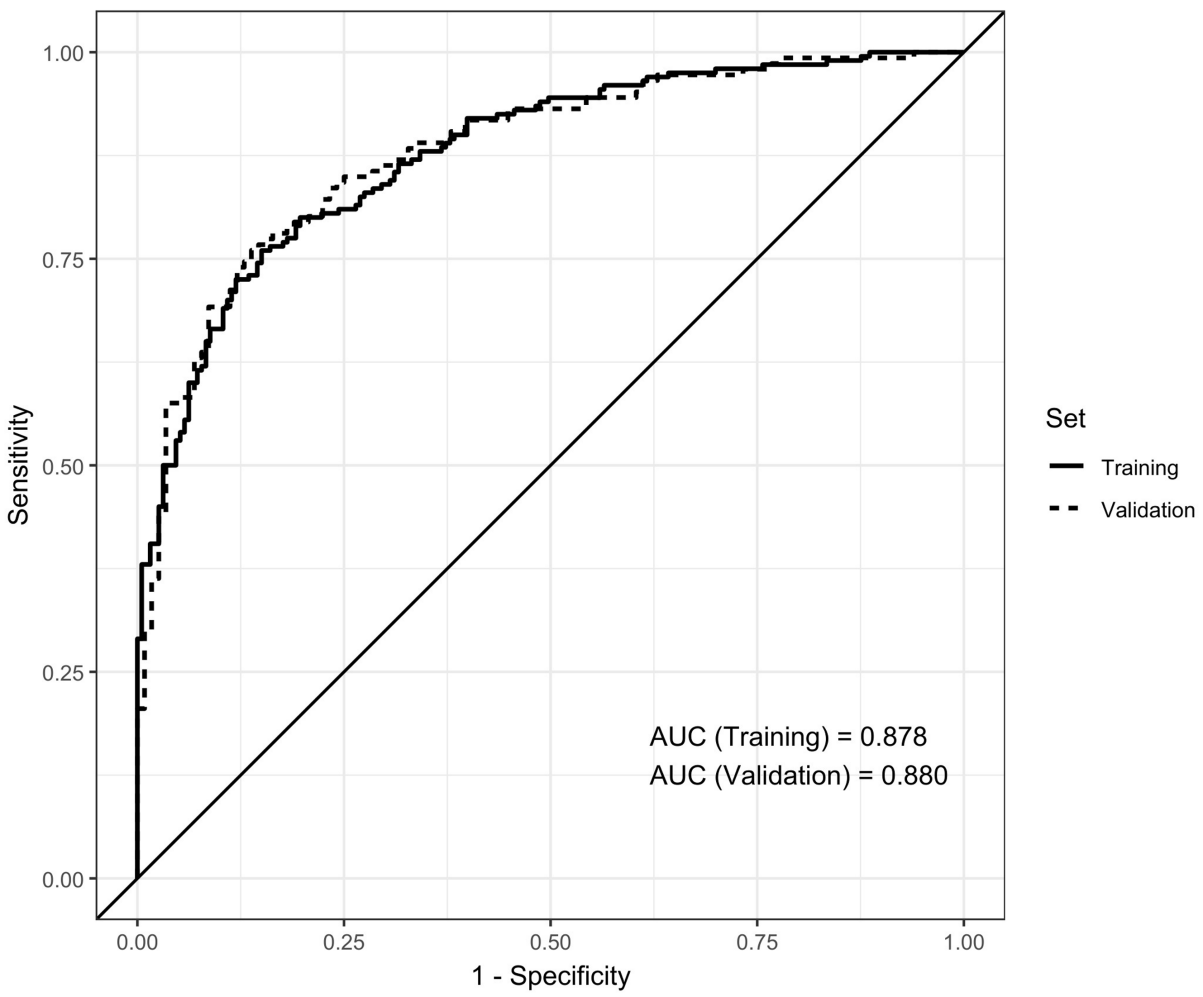
Discrimination of the nomogram in the training and test sets.

The nomogram showed good discrimination for predicting poor 3-month outcome. The C-index/AUC was 0.878 (bootstrap 95% CI: 0.846–0.908) in the training set and 0.880 (bootstrap 95% CI: 0.833–0.924) in the test set (Figure 3).

**Figure 4:**
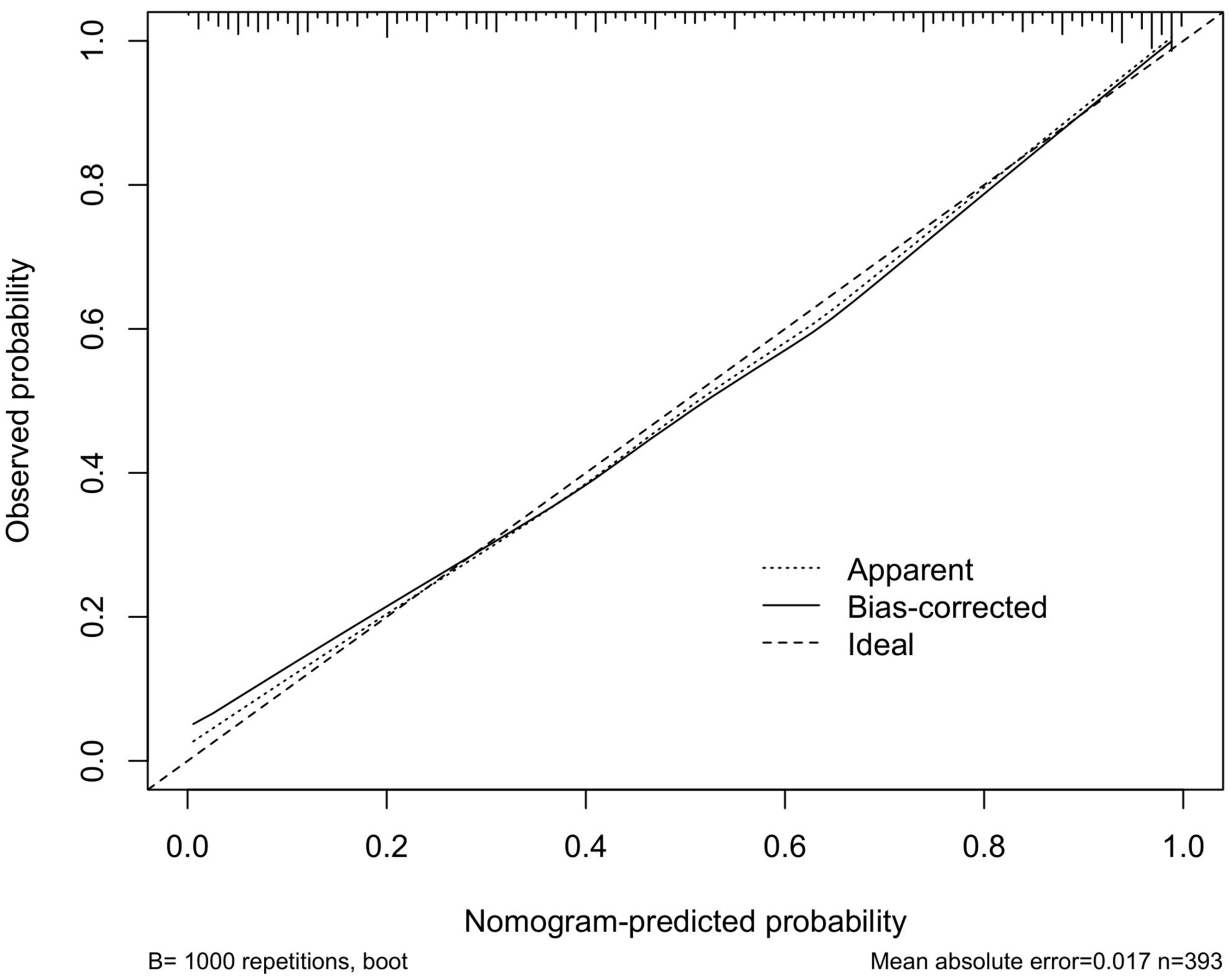
Calibration curves of the nomogram (1,000 bootstrap resamples) in the training and test sets.

Calibration curves based on 1,000 bootstrap resamples showed good agreement between predicted and observed probabilities in both datasets (Figure 4). The Hosmer–Lemeshow test did not indicate significant lack of fit in either the training set (*χ*^2^ = 7.869, df = 8, *p* = 0.446) or the test set (*χ*^2^ = 9.572, df = 8, *p* = 0.296).

**Figure 5:**
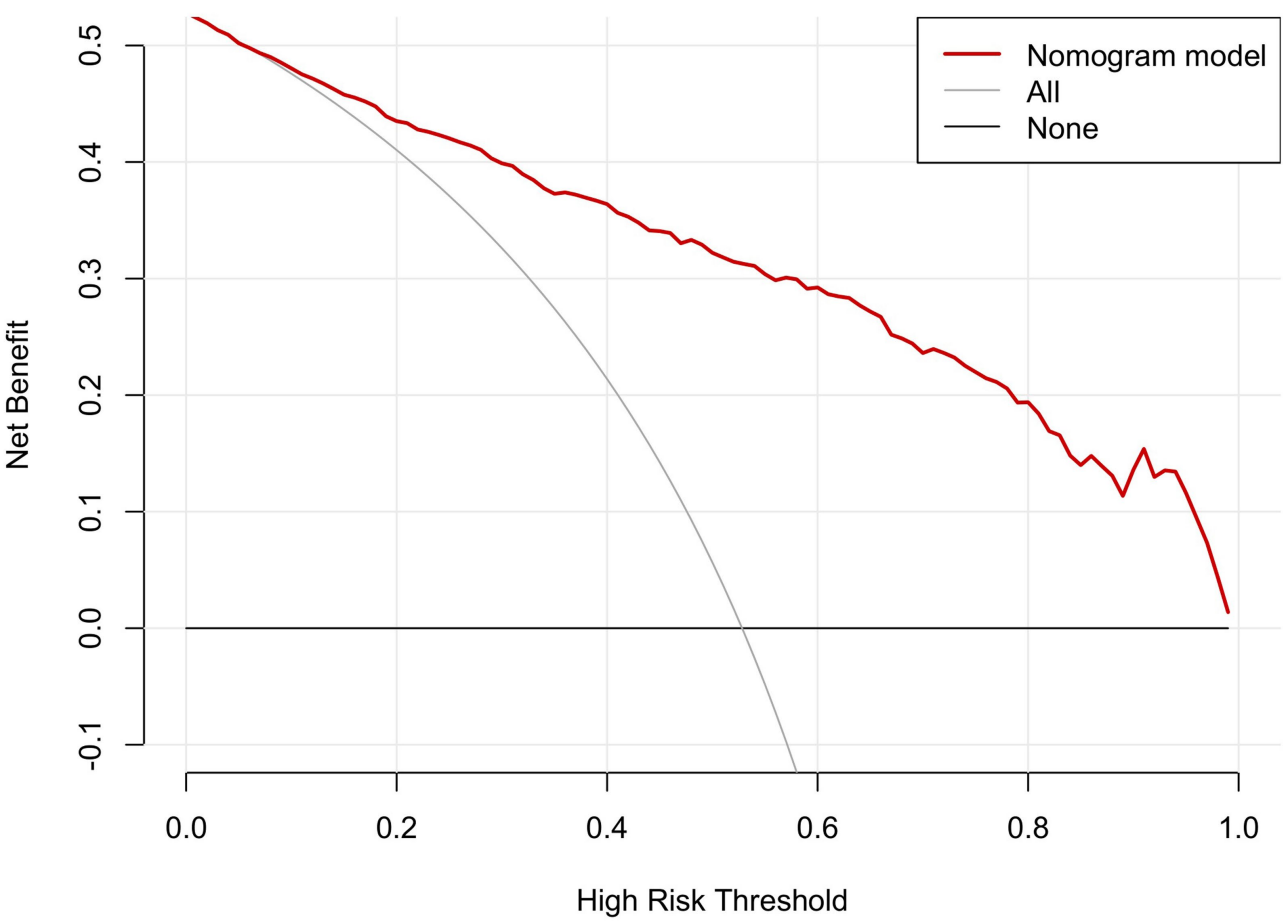
Decision curve analysis (DCA) of the nomogram for predicting poor 3-month outcome.

Decision curve analysis showed that the nomogram provided greater net benefit than treat-all and treat-none strategies across a broad range of threshold probabilities, approximately 0.07–0.98 (Figure 5).

**Figure 6:**
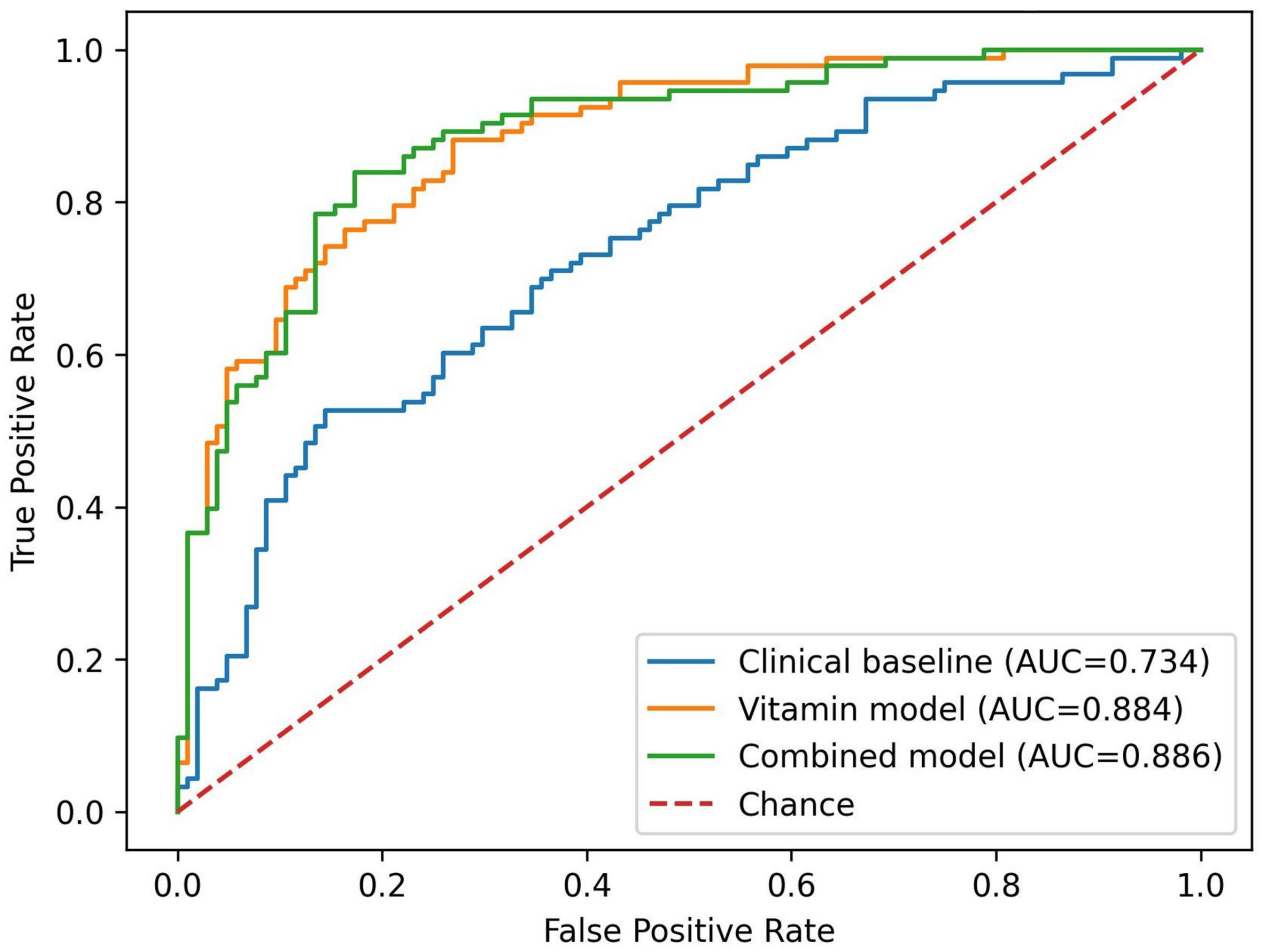
ROC curves for comparison of the clinical baseline model, vitamin-based model, and combined model in the test set (30%).

**Figure 7:**
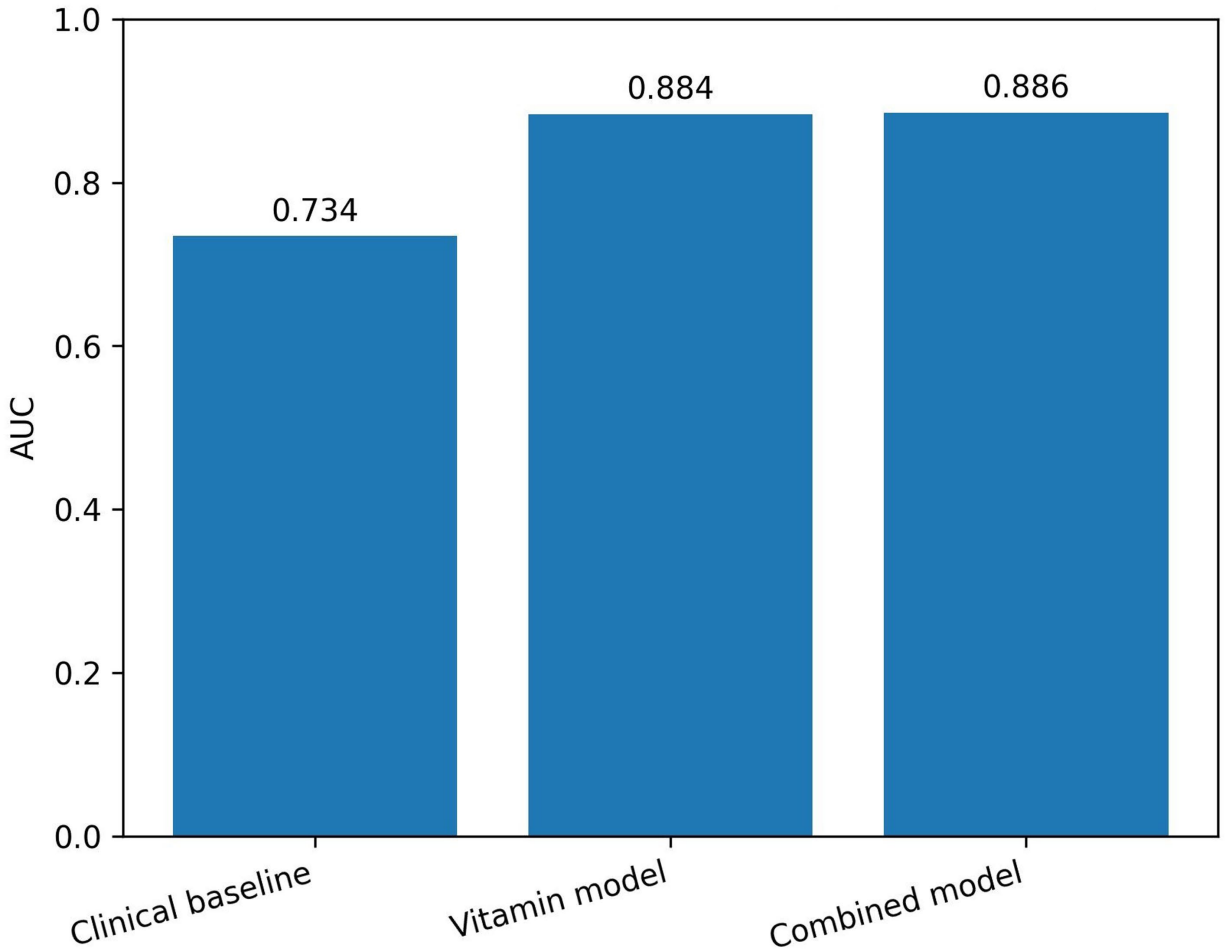
AUC comparison across the three models in the test set (30%).

**Figure 8:**
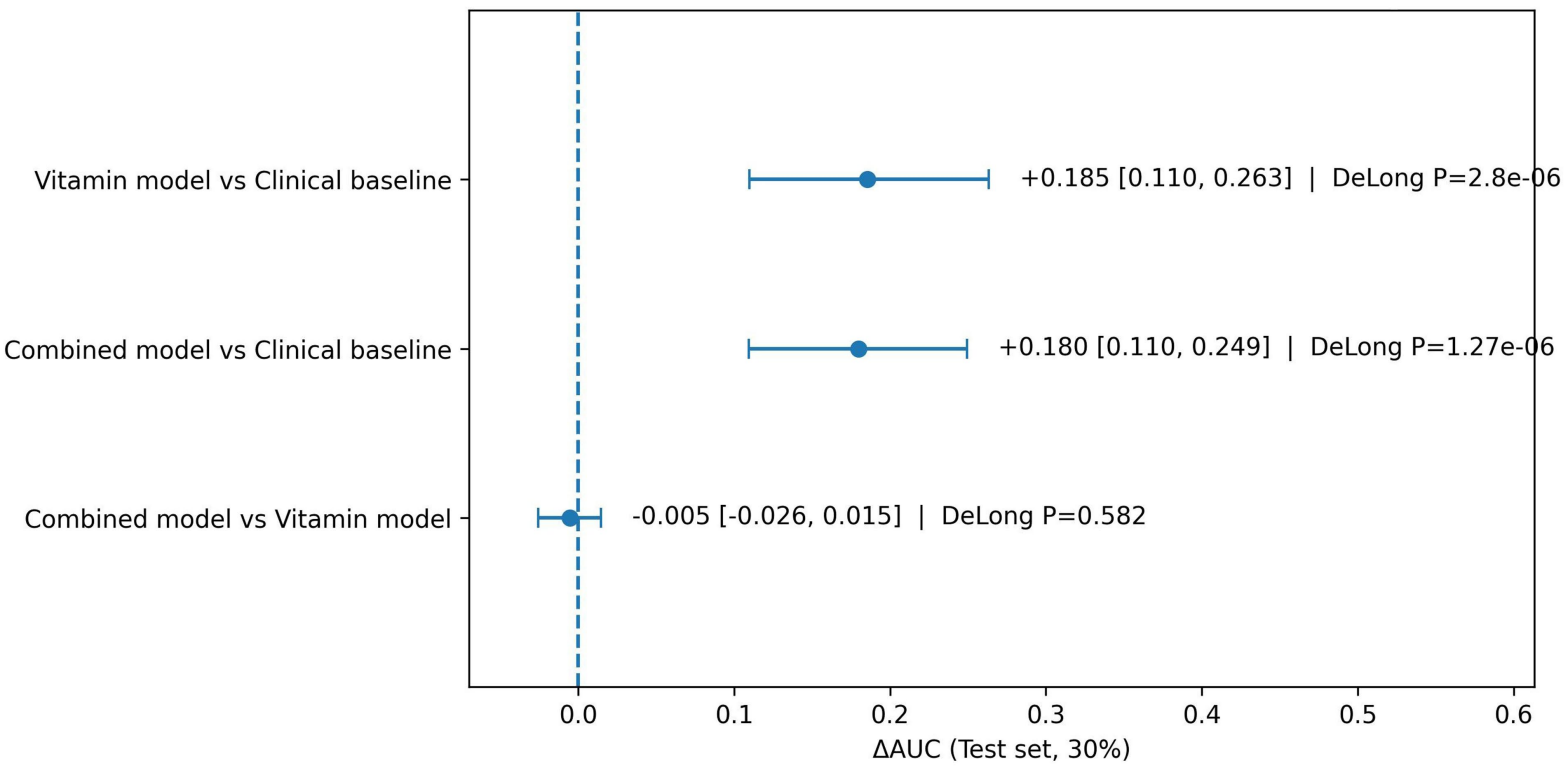
Incremental discrimination (ΔAUC) with 95% confidence intervals and DeLong *p*-values in the test set (30%).

In the held-out test set, the clinical baseline model showed moderate discrimination (AUC = 0.734), whereas the vitamin-based model (AUC = 0.884) and combined model (AUC = 0.886) performed better (Figures 6, 7). Compared with the clinical baseline model, the vitamin-based model improved discrimination by ΔAUC = 0.185 (95% CI: 0.110–0.263; DeLong *p* < 0.001), and the combined model improved discrimination by ΔAUC = 0.180 (95% CI: 0.110–0.249; DeLong *p* < 0.001). The combined model did not significantly outperform the vitamin-based model (ΔAUC = −0.005, 95% CI: −0.026 to 0.015; DeLong *p* = 0.582) (Figure 8).

## 4 Discussion

### 4.1 Main findings and context within the literature

AIS is a major cause of disability, and outcome prediction remains challenging even in the era of modern reperfusion therapy (18). In this retrospective cohort, seven vitamin-related biomarkers-vitamins D, E, A, K1, B12, folate, and homocysteine-were independently associated with 3-month functional outcome. On the basis of these markers, we developed and internally validated a nomogram that showed good discrimination, acceptable calibration, and potential clinical utility. These findings support the concept that vitamin-related biomarkers may capture prognostic information that is complementary to conventional clinical assessment (19–29).

Our results should be interpreted as predictive rather than causal. In particular, the apparently counterintuitive direction of association for vitamin D and folate may reflect acute-phase biological shifts, stress-related redistribution, pre-admission nutritional exposure, residual confounding, non-linear associations, seasonality, obesity, or other unmeasured clinical factors rather than a direct harmful effect of these micronutrients.

### 4.2 Biological plausibility and potential mechanisms

Biologically, vitamin-related biomarkers may reflect several pathways relevant to stroke recovery, including oxidative stress, inflammation, endothelial dysfunction, coagulation, and nutritional-metabolic reserve. Vitamin E may reduce lipid peroxidation and membrane injury during ischemia–reperfusion (30–32). Vitamin A is involved in neuronal differentiation and synaptic plasticity (33), and vitamin K1 may contribute to neuronal protection in addition to its role in coagulation (34). Folate and vitamin B12 participate in homocysteine metabolism, whereas elevated homocysteine has been linked to endothelial dysfunction, inflammation, oxidative stress, and thrombosis (35–37). Together, these markers may capture overlapping but nonidentical biological domains relevant to post-stroke recovery.

These findings do not imply that higher or lower levels of any single vitamin should directly guide supplementation decisions after stroke. Rather, the combined biomarker pattern may function as an integrated prognostic signal within this cohort. The positive association between folate and poor outcome should likewise not be interpreted as evidence of biological harm, because it may reflect residual confounding, altered handling during acute illness, or context-dependent and potentially non-linear relationships. Future studies should evaluate repeated measurements, non-linear effects, and mechanistic pathways in datasets with more detailed information on diet, supplementation, organ function, inflammation, and treatment exposure. In addition, future prognostic studies should consider patient-reported self-care ability, long-term treatment adherence, rehabilitation-related physical outcomes, and mechanistic evidence on vitamin D–related neuroprotection when evaluating post-stroke recovery and biomarker-based risk stratification (44–47).

### 4.3 Clinical implications and potential utility of the nomogram

The predictive value of any single biomarker was modest, with AUCs ranging from 0.590 to 0.789, which supports the rationale for multivariable integration rather than reliance on isolated laboratory results. The vitamin-based nomogram achieved AUCs of 0.878 in the training set and 0.880 in the test set, with favorable calibration and decision-curve performance. These findings suggest that combining multiple vitamin-related biomarkers may improve identification of patients at risk of poor 3-month outcome after AIS.

Relative to the clinical baseline model, the vitamin-based and combined models showed higher discrimination in the held-out test set. However, this apparent incremental value should be interpreted cautiously because the study was retrospective and single-center, and several potentially important confounders, including TOAST subtype, reperfusion treatment details, onset-to-treatment interval, inflammatory markers, and detailed renal or hepatic function indices, were not uniformly available for adjustment. Accordingly, the observed associations should not be interpreted as evidence that vitamin-related biomarkers are intrinsically superior to established clinical variables.

Moreover, because this was a single-center retrospective study with an internally validated nomogram only, the model’s transportability to other hospitals, regions, laboratory platforms, and treatment settings remains uncertain. External validation in independent multicenter cohorts is therefore essential before broader clinical implementation.

From a clinical perspective, the nomogram is simple to interpret and may help generate an early, hypothesis-generating estimate of risk for closer monitoring or rehabilitation planning. However, the model should not be used as a stand-alone decision tool and should not replace established clinical assessment, stroke severity evaluation, imaging findings, or physician judgment. Because only internal validation was performed, clinical use should remain exploratory until external validation, recalibration if needed, and prospective impact assessment are completed.

### 4.4 Limitations and future directions

This study has several limitations that should be emphasized. First, this was a single-center retrospective analysis from a high-volume regional stroke center, which may introduce selection bias, information bias, and center-specific practice patterns. The relatively standardized biomarker testing pathway at our center enabled retrospective extraction of vitamin-related biomarker data, but it may also limit generalizability to hospitals where such testing is not routinely performed. Therefore, the novelty and transportability of the findings should be considered limited until the model is externally validated in independent centers with different laboratory platforms and patient populations.

Second, although we attempted to improve statistical modeling and confounding control by considering available clinical covariates and comparator models, several important variables were not uniformly available in the retrospective database, including TOAST subtype, detailed reperfusion treatment information, onset-to-treatment interval, inflammatory markers, renal and hepatic function indices, dietary intake, seasonality, and pre-admission supplementation history. Residual confounding and reverse causation therefore cannot be excluded, and the associations should be interpreted as predictive rather than causal.

Third, vitamin-related biomarkers were measured only once at baseline, so dynamic changes during the acute and recovery phases could not be evaluated. Fourth, some assay-level laboratory details were not uniformly retrievable, which may have introduced measurement variability. Finally, although the events-per-variable ratio was adequate and internal validation was performed using a held-out test set and bootstrap resampling, overfitting cannot be completely excluded without external validation. Future multicenter prospective studies should include longitudinal biomarker assessment, broader clinical covariates, external validation, recalibration, and evaluation of whether use of the model improves patient management or outcomes. More practically, future large-scale cohorts should be designed as international, longitudinal precision-medicine platforms rather than as model-validation datasets alone. First, psychological stress, immune-stress responses, motivation, adherence, sleep, behavioral activation, and caregiver or social-support indicators should be recorded, because stress-related immune pathways may influence both recovery capacity and biomarker interpretation (38). Second, preventive-care context should be documented, including whether patients are undergoing primary or secondary prevention pathways and whether modifiable risk factors are being actively managed (39). Third, rehabilitation training, physical activity, and exercise-related biomarker variation should be captured, since training status and exercise load can modify cardiovascular and metabolic biomarkers (40). Fourth, the month or season of blood sampling, latitude, sunlight exposure, diet, micronutrient intake, and supplementation should be recorded, because vitamin D status and related nutritional biomarkers may vary with seasonal exposure and training environment (41). Fifth, health-examination and preventive follow-up frameworks should be incorporated to determine whether biomarker-based risk estimates can be translated into practical screening, counseling, and intervention pathways (42). Finally, future cohorts should also consider stress-reaction concepts from preventive psychiatry, including the transition from screening to a broader somatopsychic understanding of risk, when selecting behavioral and psychosocial markers for precision prevention (43). Incorporating these variables would help clarify whether the biomarker score reflects nutritional-metabolic reserve, acute stress biology, behavioral recovery capacity, preventive-care engagement, or residual confounding. Future studies should further evaluate whether vitamin-related risk stratification can guide targeted preventive strategies, such as individualized nutritional assessment, supplementation review, rehabilitation intensity adjustment, behavioral support, adherence enhancement, and follow-up planning. In this way, biomarker-based prediction models may move beyond statistical risk estimation toward clinically actionable precision prevention after AIS.

## 5 Conclusion

This vitamin biomarker-based model may provide a preliminary and hypothesis-generating framework for early risk stratification after AIS. Given the single-center retrospective design, incomplete adjustment for several potentially important confounders, and absence of external validation, the model should currently be considered exploratory and should not be used for routine clinical decision-making before validation in independent multicenter cohorts.

## Data Availability

The raw data supporting the conclusions of this article will be made available by the authors, without undue reservation.
