# Supplementary material for: A vitamin-biomarker risk score for 90-day functional outcome after acute ischemic stroke: development and internal validation in a retrospective cohort
Source: Front Neurol. 2026 May 29;17:1784930. doi: 10.3389/fneur.2026.1784930 (PMC13267819; doi:10.3389/fneur.2026.1784930)
Supplement: Supplementary file 1 [file Table_1.DOCX]

**Supplementary Table 1. Key net benefit values at common thresholds**

| **Threshold** | **NB_model** | **NB_treat_all** | **NB_treat_none** | **NB_model_minus_all** | **NB_model_minus_none** |
| --- | --- | --- | --- | --- | --- |
| 0.050 | 0.502 | 0.503 | 0.000 | -0.002 | 0.502 |
| 0.100 | 0.480 | 0.476 | 0.000 | 0.004 | 0.480 |
| 0.150 | 0.459 | 0.445 | 0.000 | 0.014 | 0.459 |
| 0.200 | 0.435 | 0.410 | 0.000 | 0.024 | 0.435 |
| 0.300 | 0.398 | 0.326 | 0.000 | 0.072 | 0.398 |
| 0.500 | 0.321 | 0.056 | 0.000 | 0.264 | 0.321 |

Notes: Outcome event was defined as poor prognosis (mRS > 2 at 3 months). Net benefit (NB) was calculated using decision curve analysis based on predicted probabilities from the nomogram model (logistic regression with serum biomarkers: homocysteine, folate, vitamin B12, vitamin K1, vitamin A, vitamin E, and vitamin D).

**Key model performance metrics (computed from the provided dataset):**

• C-index (AUC) in training set: 0.878; in test set: 0.880 (70/30 split, stratified; random seed=322).

• Hosmer–Lemeshow test (g=10) in training set: χ²=7.869, df=8, P=0.446.

• Hosmer–Lemeshow test (g=10) in test set: χ²=9.572, df=8, P=0.296.

• DCA clinical net-benefit interval: the model showed higher net benefit than both ‘treat-all’ and ‘treat-none’ at threshold probabilities approximately 0.07–0.98 (step=0.01).

• Hosmer–Lemeshow test (g=10) in the full cohort model: χ²=3.455, df=8, P=0.903.
